# First genomic insights into the introgression of almond PPV-Marcus resistance into peach

**DOI:** 10.3389/fpls.2026.1920650

**Published:** 2026-08-18

**Authors:** Lucía Rodríguez-Robles, Pedro Martínez-Gómez, Jorge Mas-Gómez, Pedro José Martínez-García, Manuel Rubio

**Affiliations:** Department of Plant Breeding, Centro de Edafología y Biología Aplicada del Segura (CEBAS-CSIC), Murcia, Spain

**Keywords:** almond, linkage mapping, peach, plum pox virus, *Prunus*, QTL, sharka

## Abstract

**Aim:**

Sharka, caused by *Plum pox virus* (PPV), is one of the most damaging viral diseases of stone fruit crops, with peach among the most susceptible cultivated *Prunus* species. Almond is a promising source of resistance, but its genetic architecture and expression in a peach genetic background remain largely unknown. This study aimed to construct parental genetic linkage maps and identify genomic regions associated with PPV response in almond × peach interspecific populations.

**Methods:**

Progenies derived from the almond cultivars ‘Del Cid’, ‘Garrigues’, and ‘Mono’ were evaluated by RT-PCR after graft inoculation with the PPV-Marcus (PPV-M) strain over consecutive infection cycles. Phenotypic data were summarized for each genotype using best linear unbiased estimates (BLUEs). High-density SNP almond and peach arrays were used to construct parental maps for ‘Garrigues’ and ‘Mono’ and perform quantitative trait locus (QTL) analysis.

**Results:**

Phenotypic variation was observed among and within families. ‘Del Cid’-derived progenies showed the greatest resistance, ‘Garrigues’-derived progenies displayed intermediate responses, and ‘Mono’-derived progenies showed greater susceptibility and variability. The parental maps covered 546.69 cM in ‘Mono’ and 521.72 cM in ‘Garrigues’, with average intervals of 0.61 and 1.26 cM per unique marker position, respectively, and showed strong collinearity with the reference genome. QTL associated with PPV-M response were detected on linkage groups (LG) 1 and 6 in ‘Garrigues’ and LG2 in ‘Mono’. The main QTL in ‘Garrigues’ peaked near 22.39 Mb on LG1, whereas the ‘Mono’ QTL was located at 22.27–22.62 Mb on LG2; a weaker QTL was detected near 25.38 Mb on LG6 in ‘Garrigues’. The results support a quantitative and genetic-background-dependent architecture of PPV resistance.

**Conclusion:**

This study provides the first evidence of genomic regions associated with PPV-Marcus response in almond × peach populations. The detected QTLs provide an initial basis to support the introgression of almond-derived resistance into peach breeding material.

## 1 Introduction

Peach is one of the most important stone fruit (*Prunus*) species around the world, with an annual production of more than 27 million tons (Mt) in 2023 (FAOSTAT, 2026). Peach production is threatened by various pests and diseases, among which sharka is considered one of the most devastating diseases affecting stone fruit trees worldwide (Scholthof et al., 2011; Rubio et al., 2017). This disease is caused by Plum pox virus (PPV), a positive-strand RNA virus of the *Potyviridae* family (García et al., 2014). In susceptible genotypes, PPV infection can induce chlorotic rings, mosaics and vein clearing in leaves, as well as fruit deformation, discoloration, necrotic lesions and premature fruit drop, resulting in substantial yield and quality losses (García et al., 2014; Rubio et al., 2017, 2021). PPV comprises several genetically and biologically distinct strains, among which the Marcus strain (PPV-M) is particularly relevant to peach production because of its high adaptation to this host and its association with rapid epidemics and efficient aphid-mediated transmission (García et al., 2014, 2025). The global cost of sharka damage is estimated to have exceeded €13 billion over the past century, including direct costs such as yield losses, eradication and disease-control programs, border rejections, and compensation measures, as well as indirect costs associated with prevention, diagnostics, trade restrictions, and research (Cambra et al., 2024). Given this economic and phytosanitary impact, understanding PPV response in peach and identifying sources of resistance are key priorities for breeding programs.

In this context, natural sources of PPV resistance are scarce in cultivated peach (*Prunus persica* L. Batsch), and most peach cultivars are susceptible to infection. Therefore, related *Prunus* species have been investigated as potential donors of resistance for peach breeding. Quantitative resistance has been identified in the wild peach relative *Prunus davidiana*, although its transfer to peach may be influenced by the recipient genetic background (Decroocq et al., 2005; Rubio et al., 2010). Almond (*P. dulcis* [Mill.] D.A. Webb), a closely related and sexually compatible species, represents another promising source of resistance. Several almond cultivars have shown resistance to PPV under controlled conditions, and this resistance has been successfully transmitted to peach × almond interspecific hybrids (Rubio et al., 2003; Martínez-Gómez et al., 2004; Rubio et al., 2025). Moreover, grafting the resistant almond cultivar ‘Garrigues’ onto the highly susceptible peach genotype ‘GF305’ reduced PPV symptom development and viral accumulation and, when performed before inoculation, prevented the establishment of infection (Rubio et al., 2013, 2021). These results suggest that almond contains genetic and/or mobile molecular factors capable of restricting PPV multiplication or movement in a peach background (Rubio et al., 2013, 2021). Thus, peach × almond interspecific hybridization could provide a valuable strategy for introducing almond-derived resistance into peach while generating novel genetic variability to identify and select resistance-associated loci.

The genetic architecture of PPV resistance has been investigated through linkage mapping, QTL analysis and genome-wide association studies in several *Prunus* species. Studies using interspecific populations derived from crosses between peach and resistant *P. davidiana* clone P1908 identified polygenic resistance, controlled by several QTLs distributed across different linkage groups (Decroocq et al., 2005; Marandel et al., 2009a; Rubio et al., 2010). Some of these QTL regions contained candidate genes potentially involved in plant-virus interactions, including resistance gene analogues and host translation initiation factors required by potyviruses for infection (Decroocq et al., 2005; Marandel et al., 2009a). In apricot, a major resistance region known as *PPVres* has repeatedly been mapped to linkage group 1, and genes encoding meprin and TRAF homology domain proteins have been proposed as candidates related to this resistance (Marandel et al., 2009b). Nevertheless, markers linked exclusively to PPVres do not accurately predict resistance across all genetic backgrounds, indicating that additional loci, epistatic interactions and genotype-dependent effects contribute to the resistant phenotype (Decroocq et al., 2014; Mariette et al., 2016). These findings emphasize the importance of evaluating PPV response in different genetic backgrounds and over multiple experimental cycles. In this context, linkage mapping and QTL analysis in peach × almond interspecific populations could reveal almond-derived genomic regions associated with PPV resistance.

Despite advances in identifying PPV-M resistance loci across *Prunus*, the genetic basis of almond-derived resistance and its expression in a peach genetic background remain unclear. Therefore, we evaluated sharka resistance in interspecific almond × peach progenies under controlled conditions over three infection cycles, genotyped the populations using two high-density SNP arrays developed for almond and peach, constructed parental genetic linkage maps, identified genomic regions associated with PPV resistance-related traits, and assessed the effects of the detected QTLs across evaluation periods.

## 2 Materials and methods

**Figure 1:**
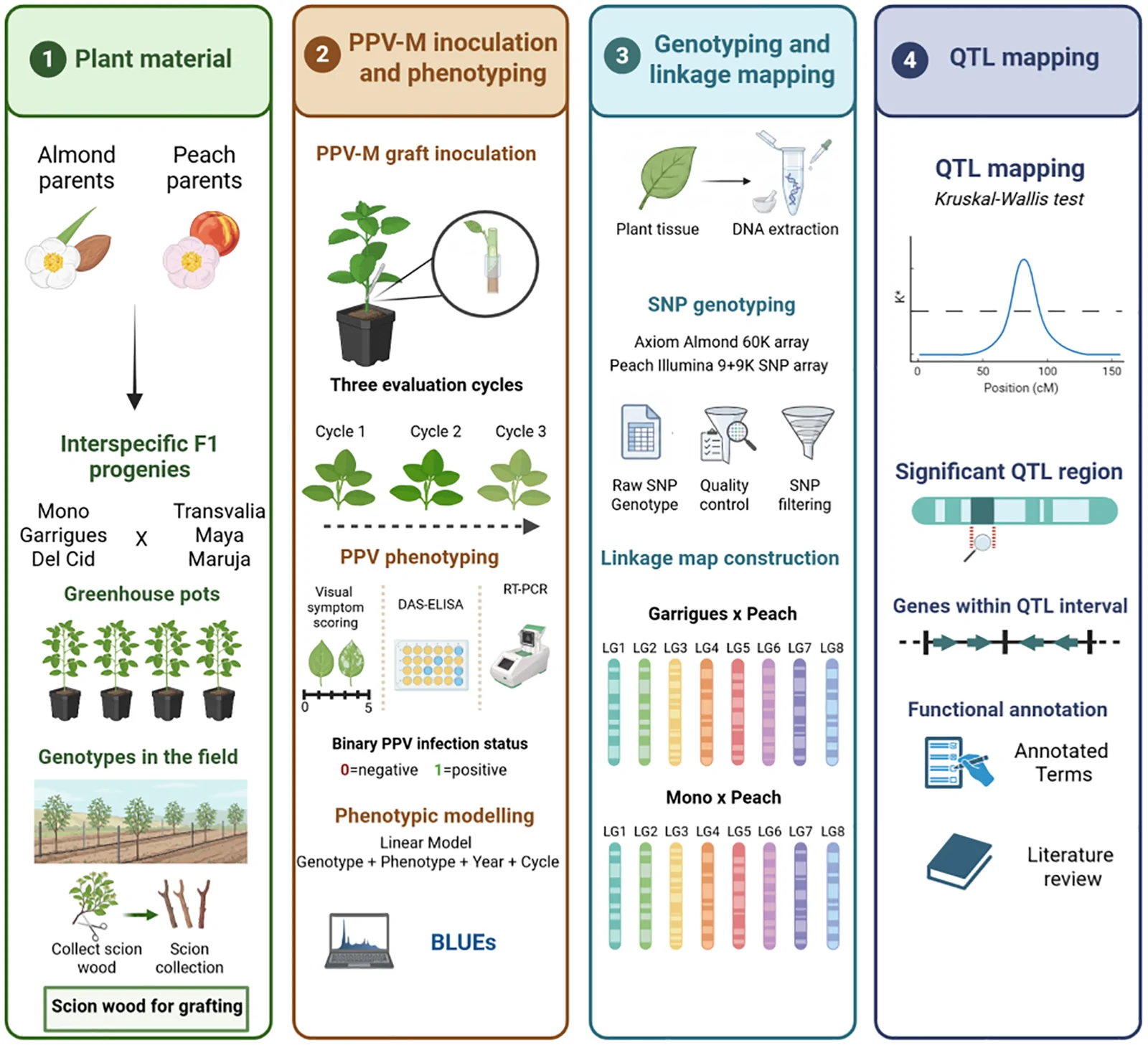
Overview of the experimental design and analytical workflow used to investigate PPV-M resistance in interspecific almond × peach populations. (Created with BioRender.com).

An overview of the experimental design and analytical workflow is presented in **Figure 1**.

### 2.1 Plant material

The plant material used in this study consisted of 110 interspecific individuals from controlled crosses between three almond cultivars, ‘Mono’, ‘Garrigues’ and ‘Del Cid’ used as female parents, and three peach cultivars, ‘Transvalia’, ‘Maya’, and ‘Maruja’ used as male parents. Sixty-two individuals were obtained from crosses involving ‘Mono’ as the female parent, including 31 individuals from ‘Mono × Transvalia’, 24 from ‘Mono × Maya’, and 7 from ‘Mono × Maruja’. Forty-eight individuals were obtained from crosses involving ‘Garrigues’ as the female parent, including 19 individuals from ‘Garrigues × Transvalia’, 16 from ‘Garrigues × Maya’, and 13 from ‘Garrigues × Maruja’. Nineteen individuals were obtained from crosses involving ‘Del Cid’ as the female parent, including eight individuals from ‘Del Cid × Transvalia’, seven from ‘Del Cid × Maya’, and four from ‘Del Cid × Maruja’. These interspecific populations were grown in 3.5 L pots in a controlled-conditions greenhouse, at the experimental farm of the ‘Centro de Edafología y Biología Aplicada del Segura’ (CEBAS-CSIC), located in Santomera, Murcia, Spain. The three almond parental genotypes were selected based on their resistance to PPV-M (‘Mono’ and ‘Del Cid’ resistant and ‘Garrigues’ tolerant), while the three peach genotypes were chosen for their contrasting agronomic and genetic characteristics to maximize segregation and facilitate linkage map construction.

### 2.2 PPV-Marcus resistance phenotyping

GF305 peach seedlings, used as biological indicators for PPV detection (Bernhard et al., 1969), were obtained by stratifying seeds at 7 °C for 12 weeks. Germinated seeds were sown in pots and grown for two months, then inoculated by grafting a piece of bark from infected GF305 seedlings previously inoculated with the PPV Marcus isolate “KZM-RM.1” (GenBank: PX549134; Rodríguez-Robles et al., 2026) and showing severe sharka symptoms. One month after infection, one bud from each interspecific hybrid genotype under evaluation was grafted onto the infected GF305 rootstock (Rubio et al., 2013). Each genotype was evaluated using at least three biological replicates, subject to plant survival. To promote symptom expression and enhance viral replication, plants were subjected to controlled artificial growth cycles consisting of alternating dormancy and active growth periods (Rubio et al., 2013). Dormancy was induced twice yearly (December-January and July-August) by maintaining plants in a cold chamber at 7 °C in complete darkness for two months. Subsequently, plants were transferred to greenhouse conditions to resume growth during the artificial spring periods (February–June and September–November). A total of three phenotyping cycles were conducted to determine the behavior of each descendant against PPV-M.

In each cycle, the behavior assessment was conducted eight to ten weeks after transfer to the greenhouse using visual symptom scoring, followed by a double-antibody sandwich indirect ELISA (DASI-ELISA) being the two methods used as preliminary screening tools, while reverse transcription polymerase chain reaction (RT-PCR) was considered the definitive criterion to confirm PPV-M infection status. Sharka symptom severity was visually scored at the whole-plant level using a scale from 0 to 5, where 0 indicated the absence of symptoms and 5 corresponded to maximum symptom severity (**Supplementary Figure 1**). The DASI-ELISA test was performed utilizing a PPV-specific monoclonal antibody 5B-IVIA/AMR (Plant Print Diagnostic SL, Valencia, Spain). The presence of PPV-M in leaf samples was verified by one-step RT-PCR using a universal forward primer (P1: 5’-ACCGAGACCACTACACTCCC-3’) and a strain-specific reverse primer for PPV-M (5’-CTTCAACAACGCCTGTGCGT-3’). Reactions were performed in a final volume of 25 µL containing 1× GoTaq buffer, 1.5 mM MgCl_2_, dNTPs, Triton X-100, formamide, avian myeloblastosis virus reverse transcriptase (AMV RT), GoTaq^®^ Flexi DNA polymerase (Promega, Madison, WI, USA), and both primers at a final concentration of 0.8 µM. Reverse transcription was carried out at 42 °C for 45 min, followed by an initial denaturation at 92 °C for 2 min. PCR amplification consisted of 35 cycles of denaturation at 92 °C for 30 s, primer annealing at 60 °C for 30 s, and extension at 72 °C for 60 s, followed by a final extension step at 72 °C for 10 min. Amplification products were separated by electrophoresis on 1.5% agarose gels in 1× TAE buffer and visualized after Gel Red^®^ staining (Biotium, Fremont, CA, USA). Samples yielding the expected 198-bp amplicon were considered positive for PPV-M infection.

RT-PCR results were recorded as binary phenotypic data, where each observation was scored as 0 for absence and 1 for presence of infection. To obtain a single adjusted phenotypic value per genotype for downstream genetic analyses, best linear unbiased estimates (BLUEs) were calculated using a linear model including genotype, year, and evaluation cycle as fixed effects. The resulting adjusted genotypic means accounted for environmental and temporal variation associated with year and evaluation cycle and were subsequently used as quantitative phenotypic values for quantitative trait locus (QTL) mapping. Higher BLUE values therefore indicate a greater frequency of infection (i.e., greater susceptibility), whereas lower values indicate greater resistance.

### 2.3 Genotyping

Young leaf samples were collected from each individual, and genomic DNA was extracted using a modified CTAB method adapted for plant tissues (Tong et al., 2012). DNA quality and concentration were determined using NanoDrop 2000 spectrophotometry (Thermo Fisher Scientific, Wilmington, DE, United States). F1 populations and their parental genotypes were genotyped using two high-density SNP platforms: the peach Illumina 9 + 9K SNP array (Verde et al., 2012) and the almond Axiom™ 60K SNP array (Duval et al., 2023). Raw intensity data obtained from the peach SNP platform were processed with the Genotyping Analysis Module implemented in GenomeStudio™ v2.0.5 (Illumina Inc., San Diego, CA, USA) using standard calling settings. Marker quality assessment and SNP classification were subsequently performed using ASSIsT v1.02 (Di Guardo et al., 2015) and loci assigned to the categories “Monomorphic”, “Failed”, and “NullAllele-Failed” were excluded from further analyses. For the almond SNP array, SNP dosage scores were estimated using functions from the fitPolyTools extension of the fitPoly v.3.0.0 R package, following the quality-control workflow described by Voorrips et al. (2011). Markers were retained when the overall quality parameter qall_mult was greater than 0.05. Following genotype calling and platform-specific filtering, datasets from both SNP arrays were integrated into a single combined matrix. The resulting high-quality SNP dataset was subsequently used for linkage map construction and downstream genetic analyses.

### 2.4 Linkage maps

Parental genetic linkage maps were developed for the almond parents ‘Mono’ and ‘Garrigues’ following a pseudo-testcross mapping strategy. ‘Del Cid’ was excluded due to the low number of individuals. Only informative markers were considered: those heterozygous in the corresponding almond parent and homozygous in peach. Map construction was performed using JoinMap v5 (Van, 2018). Linkage groups were established based on recombination frequencies and logarithm of odds (LOD) thresholds and subsequently assigned to the eight *Prunus* chromosomes. Recombination frequencies were converted into genetic distances (cM) using the Kosambi mapping function, applying a maximum recombination fraction threshold of 0.45. Segregation distortion was assessed for each marker by chi-square (χ²) goodness-of-fit tests against the expected Mendelian segregation ratios. Markers exhibiting significant distortion (*p-value* ≤ 0.01) were removed before final map construction (Martínez-García et al., 2013). Collinearity with the genome was assessed by comparing genetic and physical marker positions using the ‘Texas’ almond genome v2 reference assembly (Alioto et al., 2020). To obtain physical positions, SNP markers included in the linkage maps from the peach SNP array were aligned against the ‘Texas’ genome using BLAST (Camacho et al., 2009).

### 2.5 QTL analysis

QTL mapping for resistance to the Marcus strain was performed using MapQTL version 7.0 (Van Ooijen, 2023). For each marker, the Kruskal–Wallis statistic (K) was computed to test for significant differences in phenotypic distributions among genotype classes. Because symptom severity data did not follow a normal distribution, a non-parametric framework was considered appropriate for QTL detection. Markers showing significant associations (*p-value* ≤ 0.05) were considered indicative of putative QTL. Genes located within the most significant QTL intervals were examined, and their functional annotations were retrieved from the Genome Database for Rosaceae (GDR) (Jung et al., 2018).

## 3 Results

### 3.1 PPV-Marcus resistance phenotyping

**Figure 2:**
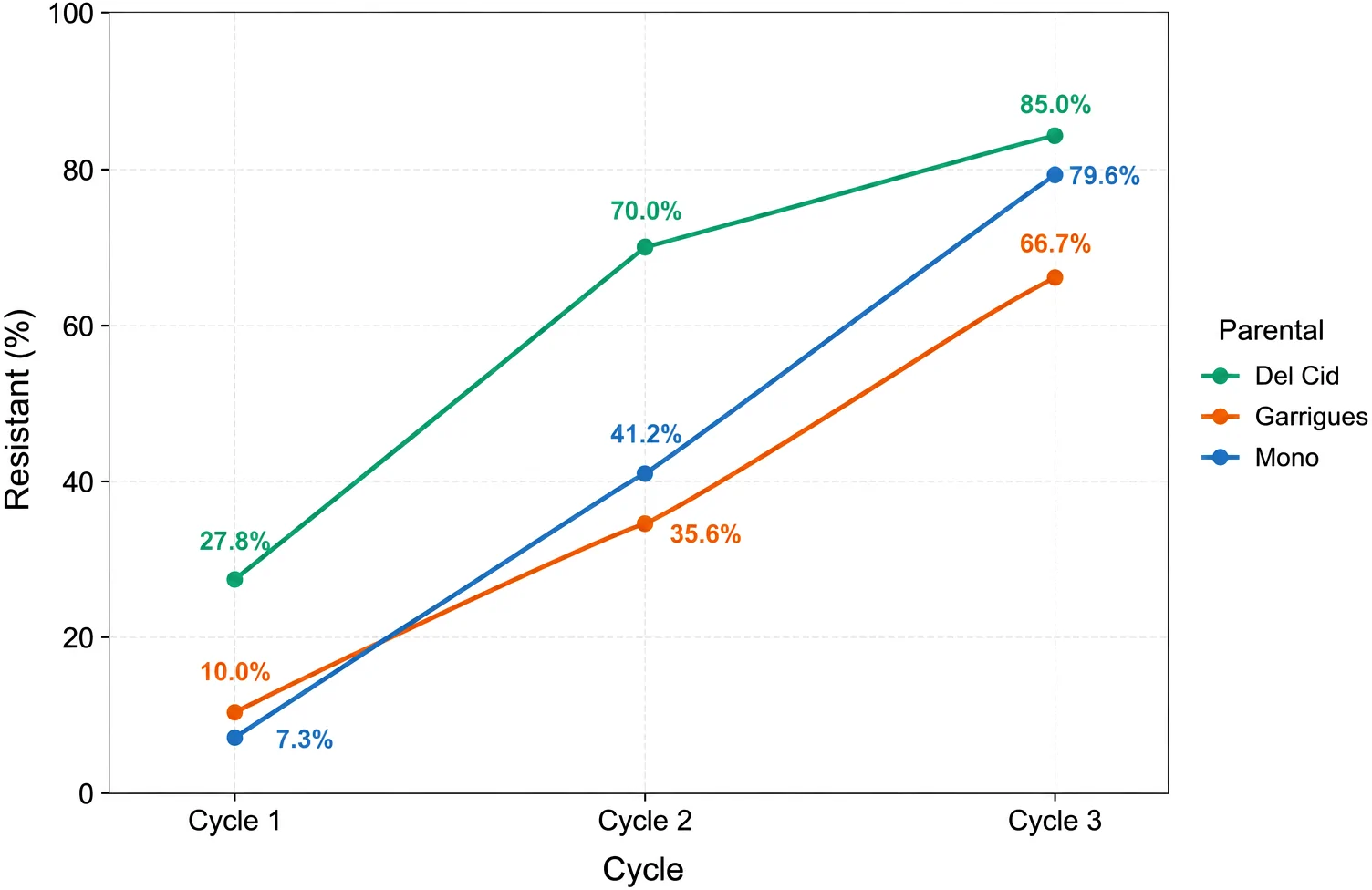
Genotypes were classified as infected when at least one biological replicate tested positive.

Preliminary phenotyping methods were also employed; however, the plants showed few or no symptoms, with almost all individuals receiving a symptom score of 0. Overall, the DASI-ELISA results followed patterns similar to those obtained by RT-PCR (data not shown). Phenotypic segregation for PPV-M resistance, based on RT-PCR results, was observed in all evaluated populations, with both resistant and susceptible individuals identified across all infection cycles (**Figure 2**). Overall, infection rates decreased as the infection cycles progressed. Families derived from the almond parental genotype ‘Del Cid’ showed the highest level of resistance, with mean resistance rates of 27.8% in cycle 1, 70.0% in cycle 2, and 85.0% in cycle 3. The family derived from parental genotype ‘Mono’ showed the second highest mean resistance level, with resistance rates of 7.3% in cycle 1, 41.2% in cycle 2, and 79.6% in cycle 3. In contrast, the family derived from parental genotype ‘Garrigues’ was the most susceptible, showing mean resistance rates of 10.0% in cycle 1, 35.6% in cycle 2, and 66.7% in cycle 3.

**Figure 3:**
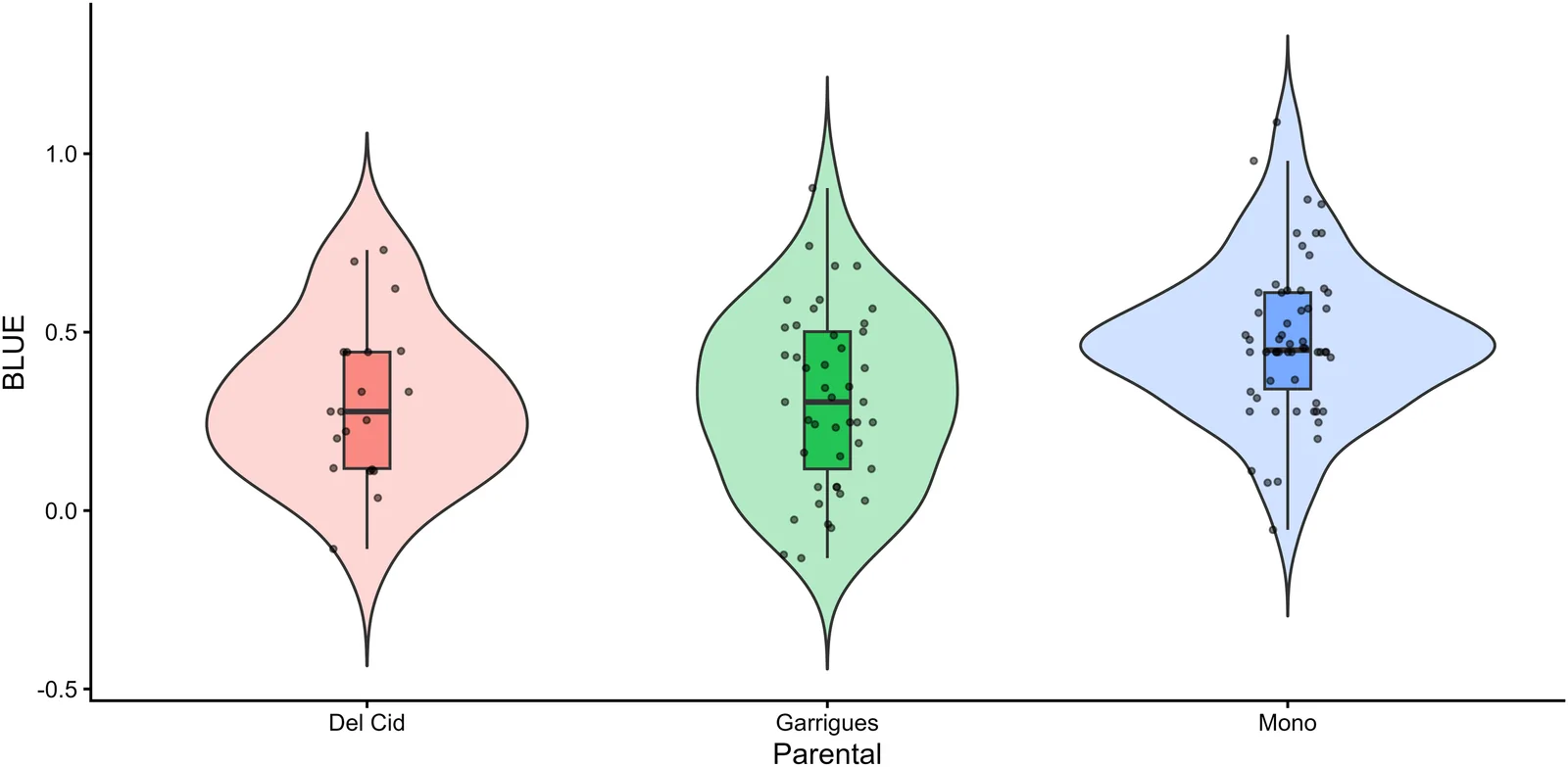
Distribution of disease-response BLUE values among progenies grouped by parental genotype. Each point represents one genotype; violin plots show the density distribution, and boxplots indicate the median and interquartile range.

BLUE values were consistent with the phenotypic assessments (**Figure 3**). Progenies derived from ‘Del Cid’ exhibited the lowest BLUE values, with a median of approximately 0.3, whereas those derived from ‘Garrigues’ showed intermediate values. In contrast, progenies derived from ‘Mono’ displayed the highest BLUE values, with a median close to 0.5. Although considerable overlap was observed among the distributions, the overall trend indicated a progressive increase in BLUE values from ‘Del Cid’ to ‘Mono’. In addition, families derived from ‘Mono’ showed greater variability and reached the highest maximum BLUE values.

### 3.2 Genotyping and SNP filtering

Genotyping of the interspecific progenies and parental genotypes was performed using both the peach and almond SNP arrays (**Supplementary Table 1**). For the peach SNP array, genotype curation classified 1,550 SNPs as failed, 3,332 as monomorphic, and 1,800 as null allele-failed markers; all of these were discarded. In contrast, 3,821 markers were selected, being 505 and 476 SNPs classified as OneHomozygRare_HWE and OneHomozygRare_NotHWE, respectively, 1,624 robust markers, and 2,928 shifted homozygous markers were retained, resulting in an initial dataset of 9,354 SNPs. After applying a minor allele frequency threshold of 0.05, 8,925 high-quality SNPs were retained for further analyses. For the almond SNP array, SNP processing and quality control were performed using the fitPoly v3.0 workflow, resulting in the selection of 28,507 SNPs. Subsequent filtering based on a minor allele frequency threshold of 0.05 yielded a final dataset of 27,662 informative SNPs for downstream analyses. The integration of both SNP arrays with the pseudo-testcross strategy, based on the selection of markers heterozygous in the almond parent and homozygous in the peach parent, resulted in final datasets of 9,356 and 8,815 SNPs for the ‘Mono’ and ‘Garrigues’ genetic maps, respectively.

### 3.3 Map construction

**Figure 4:**
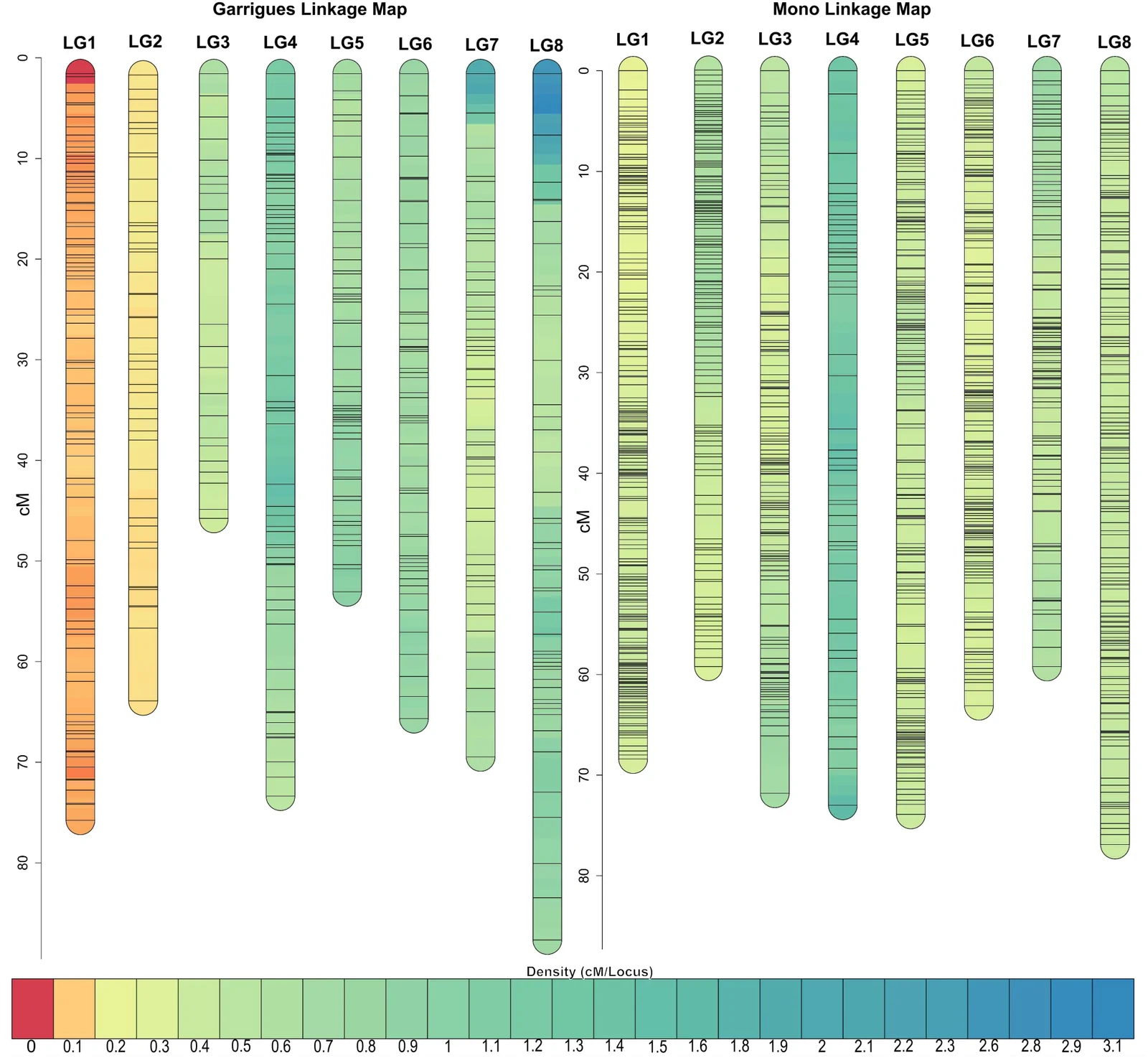
Parental genetic linkage maps showing marker distribution across the eight peach linkage groups. Genetic distances are expressed in centiMorgans (cM), and marker positions are indicated along each linkage group.

Curated genotypic data were used to construct the parental linkage maps of ‘Garrigues’ and ‘Mono’ using JoinMap. The parental ‘Garrigues’ linkage map consisted of 3,330 SNPs distributed across the eight expected linkage groups, corresponding to 444 unique genetic positions (**Table 1**). The map spanned a total genetic distance of 521.72 cM with an average marker density of 1.26 cM per unique position. Linkage group lengths varied between 44.21 cM (LG3) and 86.10 cM (LG8), while the maximum interval between adjacent markers was observed in LG2 (7.19 cM). The number of mapped SNPs per linkage group ranged from 75 markers in LG5 to 2,040 markers in LG1(**Table 1**; **Figure 4**).

**Table 1:** Summary statistics of ‘Garrigues’ and ‘Mono’ linkage maps.

LG	‘Garrigues’ parental map					‘Mono’ parental map				
	Markers	Unique position	Map length (cM)	Density (cM/unique position)	Max gap (cM)	Markers	Unique position	Map length (cM)	Density (cM/unique position)	Max gap (cM)
1	2040	88	74.24	0.84	4.25	257	235	68.36	0.29	1.93
2	692	49	61.87	1.26	7.19	141	58	60.29	1.04	2.82
3	97	26	44.21	1.70	6.45	181	159	71.83	0.45	5.73
4	81	65	71.81	1.10	7.13	58	52	73.04	1.40	6.00
5	75	52	51.49	0.99	3.11	210	187	73.94	0.40	2.48
6	89	62	64.11	1.03	2.31	219	193	63.13	0.33	1.67
7	144	56	67.89	1.21	4.47	126	112	59.16	0.53	3.24
8	112	46	86.10	1.87	6.09	204	173	76.94	0.44	1.85
Overall	3330	444	521.72	1.26	–	1396	1169	546.69	0.61	–

The ‘Mono’ parental linkage map consisted of 1,396 SNPs positioned at 1,169 unique loci, covering a total genetic distance of 546.69 cM and presenting an average marker density of 0.61 cM per unique position. Linkage group lengths ranged from 59.16 cM (LG7) to 76.94 cM (LG8). The largest inter-marker gap was detected in LG4 (6.00 cM). Marker distribution was overall balanced across linkage groups, with the number of SNPs varying from 58 markers in LG2 to 257 markers in LG1 (**Table 1**; **Figure 4**). Overall, both parental maps exhibited a clear positive association between genetic and physical positions across the eight linkage groups, supporting a high degree of collinearity with the reference genome (**Supplementary Figures 2**, **3**). The complete marker information for both maps is provided in **Supplementary Table 1**.

### 3.4 QTL mapping for PPV-M resistance

**Figure 5:**
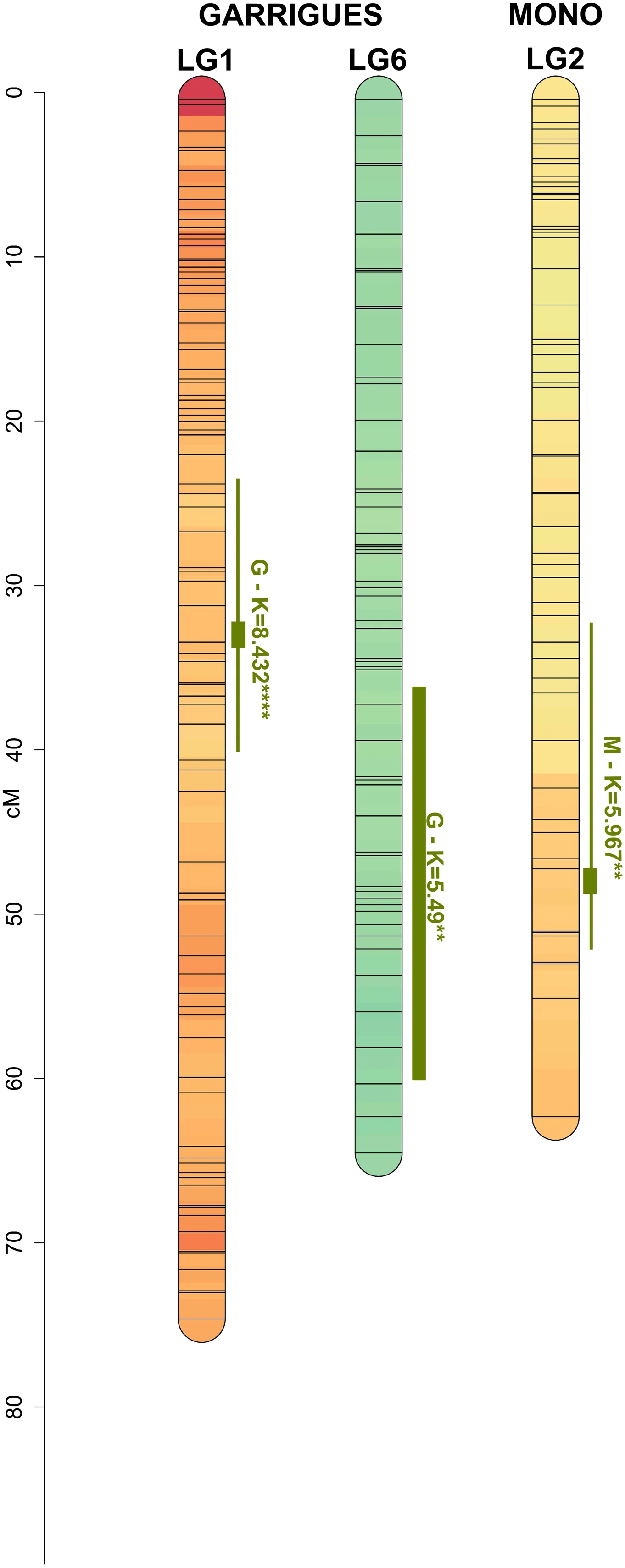
Position of the QTLs identified in the linkage map for the analyzed trait. The QTLs identified in the ‘Garrigues’ population are located on LG1 and LG6, while the QTL identified in the ‘Mono’ population is located on LG2. The vertical axis represents genetic distance in cM. Green bars indicate the QTL regions, and the labels indicate the population and QTL identifier. Asterisks indicate the maximum significance of the QTL (**p-value ≤ 0.05, ****p-value ≤ 0.005).

**Figure 6:**
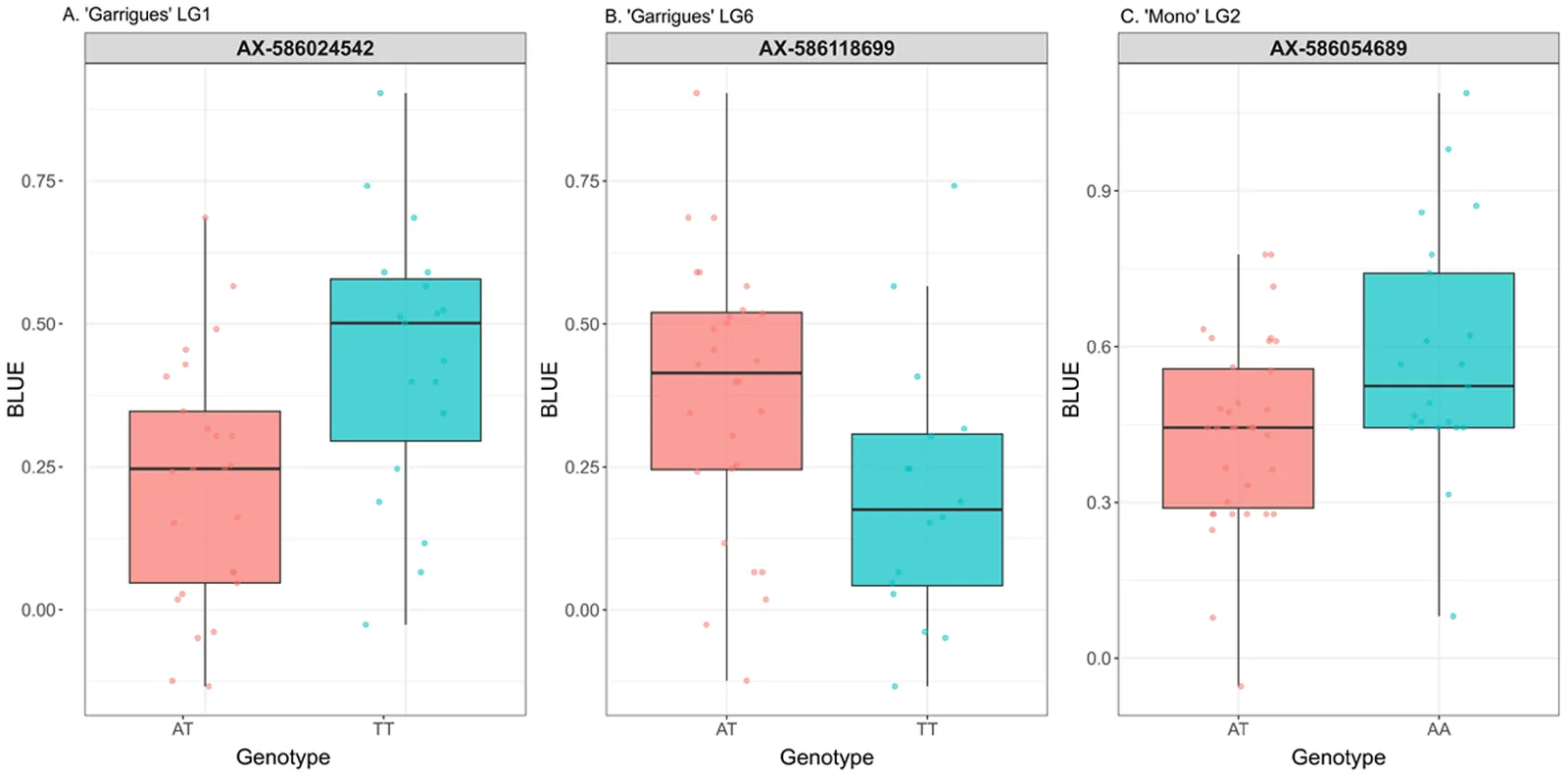
Allelic effect on PPV-M infection (susceptibility) BLUE values. Distribution of PPV-M resistance BLUE values across genotypic classes for the most significant SNPs detected. Each point represents an individual genotype, and boxplots summarize the phenotypic distribution for each allelic state.

QTL analysis was performed using BLUE values derived from PPV-M resistance RT-PCR scores. In the ‘Garrigues’ population, a QTL associated with PPV-M resistance was detected on linkage group 1 (LG1), spanning the 23.27–40.04 cM region (**Figure 5**). The strongest association with PPV-M response was detected at 32.10 cM, corresponding to marker ‘AX-586024542’ (**Figure 6A**) to ‘AX-586024896’ (K = 8.43, *p-value* ≤ 0.005), located at the physical position 22,387,301 bp. This marker showed significant differences in PPV-M resistance BLUE values between genotypic classes, with the TT genotype displaying higher BLUE values than the AT genotype (**Figure 6A**). An additional, weaker QTL was detected on LG6, spanning the 36.91–60.23 cM region (**Figure 5**). The strongest association within this region was detected near marker ‘AX-586118699’ (K = 5.49, *p-value* ≤ 0.05), corresponding to physical positions 25,384,206 bp. For marker ‘AX-586118699’, the distribution of BLUE values differed between genotypic classes, with the AT genotype showing higher median BLUE values than the TT genotype (**Figure 6B**). In the ‘Mono’ population, a single QTL associated with PPV-M resistance was detected on linkage group 2 (LG2), spanning approximately 32.9–51.1 cM. The strongest association was detected between 47.86 and 48.31 cM for markers ‘AX-586054689’, ‘AX-586055600’, ‘AX-586054755’, and ‘AX-586054663’ (K* = 5.967, p ≤ 0.05) (**Figure 5**), corresponding to a physical position range of 22,273,910–22,617,147 bp. The allelic effect plot for ‘AX-586054689’ showed higher BLUE values in the AA genotype compared with the AT genotype, supporting the effect of this genomic region on PPV-M response in the ‘Mono’ population (**Figure 6C**).

Genes with functional annotations related to plant–virus interactions were investigated within the identified QTL intervals. The ‘Garrigues’ LG1 QTL contained genes encoding a eukaryotic translation initiation factor, a ubiquitin–ribosomal protein fusion, and a 60S ribosomal protein. The ‘Mono’ LG2 QTL included genes encoding an LRR-containing receptor-like kinase and a DEAD-box RNA helicase. The broader ‘Garrigues’ LG6 QTL contained additional genes associated with RNA metabolism and immune regulation, including an RH3-like DEAD-box RNA helicase, the terminal uridylyl transferase HESO1, a NIM1-INTERACTING 1-like protein, and several WRKY70-like transcription factors (**Supplementary Table 2**).

## 4 Discussion

The use of interspecific almond × peach populations represent an important first step toward the introgression of PPV-M resistance from almond into peach and constitutes the first study to explore the genetic architecture of resistance to this strain in this genetic background. The families evaluated exhibited substantial phenotypic variation in resistance, underscoring the need to elucidate the underlying genetic architecture. The combination of high-density genotyping arrays with graft-inoculation strategies enabled the identification of the first genomic regions potentially associated with resistance to the PPV-Marcus strain in almond × peach populations.

Phenotyping for PPV-M resistance was challenging because no visual symptoms were detected, even though RT-PCR identified infected plants (Cooper and Jones, 1983; Rubio et al., 2012). This also prevented QTL mapping of symptom severity. Moreover, resistance assessment across three consecutive evaluation cycles revealed a progressive decline in infection rates as the cycles advanced, consistent with previous studies (Rubio et al., 2021, 2024). In this context, repeated evaluations across cycles (Rubio et al., 2009), together with the use of BLUEs, allowed genetic effects to be estimated while accounting for environmental variation, thereby providing a more robust quantitative assessment of resistance (Holland and Piepho, 2024). Although the phenotypic records were binary, a linear model was used as an alternative modelling approach because the logistic model generated extreme estimates with large standard errors for individuals that were consistently resistant or susceptible across all evaluation cycles. This behavior was caused by complete or quasi-complete separation (Albert and Anderson, 1984). As an internal test, individuals affected by these artifacts in the logistic model were excluded, and the individual adjusted values obtained from both approaches were found to be highly correlated. Therefore, the linear model was considered suitable for obtaining adjusted individual values while retaining all individuals, which was particularly important given the limited size of the populations.

The parental linkage maps constructed for ‘Garrigues’ and ‘Mono’ included a large number of mapped markers, resulting in high-density genetic maps spanning 521.72 and 546.69 cM, respectively. These maps were longer to other interspecific almond × peach populations such as the F_2_ interspecific (T×E) and a parental backcrossing (BC_1_) (T1E) maps reported by Donoso et al. (2015), which spanned 472.1 and 370.1 cM, respectively. In addition, the constructed maps showed similar total lengths to the high-density almond linkage map reported by Mas-Gómez et al. (2026), which spanned 509.29 cM. In terms of marker density, ‘Mono’ (0.61 cM/marker) showed a slightly lower density to T×E and T1E (0.24–0.18 cM/marker) and closer to the high-density almond map reported by Mas-Gómez et al. (2026), while ‘Garrigues’ showed the lowest density, increasing the distance per marker (1.26 cM/marker) (Donoso et al., 2015; Mas-Gómez et al., 2026). Larger gaps were also observed here in comparison to T×E and T1E of Donoso et al. (2015), the ‘Mono’ and ‘Garrigues’. These gaps and differences in marker density are generally attributable to the smaller population size compared with the populations studied by Donoso et al. (2015) (80 individuals in T×E and 190 in T1E), and Mas-Gómez et al. (2026) (114 individuals) which resulted from the scalability difficulties associated with phenotyping virus response.

The QTLs associated with PPV-M response differed between the two parental maps, with regions detected on LG1 and LG6 in the cultivar ‘Garrigues’ and on LG2 in the cultivar ‘Mono’. The LG1 QTL in ‘Garrigues’, peaking at approximately 22.39 Mb, agrees at the linkage-group level with the major PPV-resistance region repeatedly reported in apricot (Hurtado et al., 2002; Lambert et al., 2007; Soriano et al., 2008; Dondini et al., 2011). However, the classical apricot PPV resistance locus was subsequently delimited to the upper part of chromosome 1, at approximately 8.43–8.67 Mb (De Mori et al., 2019; Zuriaga et al., 2019). Therefore, although both regions are located on LG1, their different physical positions suggest that the QTL detected in ‘Garrigues’ may correspond to a distinct locus rather than to the classical PPV resistance region. The LG2 QTL detected in ‘Mono’, between approximately 22.27 and 22.62 Mb, is consistent with previous studies reporting PPV-response QTLs on LG2 in populations derived from the resistant *P. davidiana* accession P1908 (Decroocq et al., 2005). Moreover, Cirilli et al. (2017) also identified loci associated with reduced PPV susceptibility on chromosome 2 in cultivated peach, although in the upper part of LG2. Similarly, the weaker QTL detected on LG6 in ‘Garrigues’, near 25.38 Mb, agrees with previous reports of PPV-resistance QTLs on LG6 in *P. davidiana* -derived populations (Decroocq et al., 2005; Rubio et al., 2010). Overall, the QTLs detected in the interspecific populations had minor effects, suggesting that resistance is a genetically complex trait. In addition, the difference in the QTLs detected between populations may be related to the difference in the genetic background of the cultivars, with ‘Garrigues’ originating from Spain, and ‘Mono’ from the USA (Calle et al., 2025). Nevertheless, additional resistance QTLs may be identified during subsequent stages of introgression into peach (Kalluri et al., 2022). This is because resistance alleles fixed in almond but absent from peach would remain heterozygous and non-segregating in the interspecific hybrids, preventing the detection of their genetic effects. Segregation at these loci in future F_2_ or BC_1_ populations may therefore enable the identification of additional resistance genes and QTLs that could not be detected in the current populations (Kalluri et al., 2022).

The marker effects observed in the present study also showed the direction of the resistance introgression. At the LG1 QTL in ‘Garrigues’, the TT genotype at ‘AX-586024542’ showed higher PPV-response BLUE values (higher susceptibility) than the AT genotype and in the LG2 QTL of ‘Mono’, the AA homozygous genotype of ‘AX-586054689’ also showed higher BLUE values than AT. This direction of effects agrees with the introgression of resistance from almond into peach, the heterozygous classes being more resistant because they carry the almond-derived allele (the peach parent is homozygous and the almond parent contributes the alternative allele). However, an opposite pattern was observed at the weaker LG6 QTL, where the AT genotype at ‘AX-586118699’ showed higher values than TT. This finding may indicate that the almond parent carries alleles at some loci that reduce resistance. This is plausible given the polygenic architecture of complex disease-resistance traits, for which the observed phenotype results from the combined effects of multiple favorable and unfavorable alleles. Indeed, studies in wheat have shown that favorable and unfavorable resistance haploblocks are not exclusive to resistant and susceptible genotypes, respectively (Tong et al., 2024; Yadav et al., 2026). Resistant lines may still carry haplotypes that increase susceptibility, whereas moderately susceptible lines may contain unique favorable haplotypes that complement those present in resistant germplasm (Tong et al., 2024). Consequently, such susceptible lines can still serve as valuable breeding parents for stacking complementary resistance haploblocks (Yadav et al., 2026). Therefore, although marker-assisted selection is particularly effective for traits controlled by major-effect loci, genomic selection may be more appropriate for improving PPV resistance because it can capture the cumulative effects of favorable and unfavorable alleles distributed throughout the genome (Jannink et al., 2010).

Finally, genes located within the most significant QTL intervals were explored, providing preliminary insights into the biological processes that may underlie the detected QTLs. Within the ‘Garrigues’ LG1 QTL, *Prudul26A018209* encodes a putative eukaryotic translation initiation factor 4B2 (eIF4B2), and eIF4B can differentially modulate the translation of specific cellular and viral RNAs (Sanfaçon, 2015; Wang and Krishnaswamy, 2012; Mayberry et al., 2009). Although eIF4B is distinct from the eIF4E family, other translation-initiation factors, including eIF4E, eIF(iso)4E, and eIF(iso)4G, have previously been associated with PPV resistance in QTL and protein-protein interaction studies (Marandel et al., 2009a; Wang et al., 2013). Also, on LG1, *Prudul26A020456* encodes a putative ubiquitin–40S ribosomal protein S27a fusion protein (RPS27A). RPS27A is particularly relevant because viral proteins can interact with its ubiquitin moiety and disrupt ubiquitin-mediated plant defense responses (Li et al., 2019b). The ‘Mono’ LG2 QTL contains genes encoding an LRR receptor-like kinase and a DEAD-box RNA helicase, protein classes previously implicated in PPV defense, immune signaling, and RNAi-mediated antiviral immunity (Nicaise and Candresse, 2017; Li et al., 2019a; Zhang et al., 2021; Wen et al., 2024). The broad LG6 QTL identified in the ‘Garrigues’ population contains several genes previously implicated in antiviral defense, including an RH3-like DEAD-box RNA helicase, HESO1, a NIM1-like protein, and multiple WRKY70-like transcription factors. These genes are associated with RNA silencing, viral RNA turnover, and salicylic acid/NPR1-mediated immune signaling, all of which have been implicated in antiviral responses and PPV resistance (Weigel et al., 2001; Martínez-Gómez et al., 2004; Hermann et al., 2013; Rubio et al., 2021; Joly et al., 2023; Liu et al., 2023; Huang et al., 2025). Overall, these findings provide only preliminary insights into the biological processes that may underlie resistance. The detected QTL intervals remain relatively large, partly because of the limited scalability of sharka resistance evaluations. Further evidence, including gene-expression analyses and the identification of potentially disruptive mutations, will be required to elucidate the molecular mechanisms.

## 5 Conclusion

This study provides the first evidence of genomic regions associated with resistance to the PPV-Marcus strain in almond × peach interspecific populations. The construction of parental genetic maps, combined with repeated phenotypic evaluations over three phenotyping cycles, enabled the detection of QTL on LG1 and LG6 in ‘Garrigues’ and on LG2 in ‘Mono’. The relatively small effects of these QTL, the differences observed between populations and the occurrence of both favorable and unfavorable almond-derived alleles indicate that PPV-M resistance is a quantitative and genetically complex trait whose expression depends on the genetic background. The detected QTL and associated markers constitute initial targets for the introgression of PPV-M resistance into peach breeding material. The development of F_2_ and backcross populations will be particularly important for promoting the segregation of almond alleles that may have remained fixed or non-segregating in the current interspecific hybrids, potentially revealing additional resistance loci and genetic interactions. Further fine mapping and gene-expression studies could be useful to identify the causal genes and variants underlying the detected QTLs. Finally, considering the suggested genetic complexity of PPV-M resistance and the difficulties associated with its phenotyping, alternative breeding strategies, including genomic selection, should be explored for the improvement of this trait in almond breeding programs.

## Data Availability

The original contributions presented in the study are included in the article (**Supplementary Material**), further inquiries can be directed to the corresponding authors.

## Supplementary material

Supplementary Figure 1 Visual symptom severity scale used to evaluate PPV infection in GF305 peach seedlings. Representative leaf symptoms corresponding to the six severity classes used throughout the study, ranging from 0 (no visible symptoms) to 5 (very severe symptoms). Symptom scores were assigned based on the extent of chlorotic rings, mottling, leaf deformation, and overall symptom severity observed on inoculated leaves.

Supplementary Figure 2 Collinearity between genetic and physical positions of SNP markers mapped in the ‘Garrigues’ parental linkage map of the interspecific almond × peach population. Physical positions were based on the Prunus dulcis ‘Texas’ v2.0 reference genome. Each panel shows the relationship between marker physical position (Mbp) and genetic position (cM) for the eight linkage groups.

Supplementary Figure 3 Collinearity between genetic and physical positions of SNP markers mapped in the ‘Mono’ parental linkage map of the interspecific almond × peach population. Physical positions were based on the Prunus dulcis ‘Texas’ v2.0 reference genome. Each panel shows the relationship between marker physical position (Mbp) and genetic position (cM) for the eight linkage groups.
